# Alpha-tocopherol: looking beyond an antioxidant

**Published:** 2009-04-23

**Authors:** Kaya Nusret Engin

**Affiliations:** Bağcılar Education and Research Hospital, Department of Ophthalmology, Istanbul, Turkey

## Abstract

Vitamin E is an important natural antioxidant, and its most common and biologically active form is α-tocopherol. In addition to this, specific regulatory effects of vitamin E have been revealing. The body exerts a certain effort to regulate its tissue levels with specific tocopherol transport proteins and membrane receptors. Antiproliferative and protein kinase C-supressing effects of alpha-tocopherol have been previously demonstrated, which have not been mimicked by beta-tocopherol or probucol. Protein kinase C promises to be an important area of interest in the means of glaucoma and cataractogenesis. It has been shown in different models that retinal vasculer dysfunction due to hyperglycemia could be prevented by alpha-tocopherol via the diachylglycerol-protein kinase C pathway. Glutamate transporter activity has been shown to be modulated by protein kinase C. This pathway is also important in intraocular pressure-lowering effects of prostaglandin and its analogs in glaucoma therapy. Filtran surgery became another possible area of usage of alpha-tocopherol since its antiproliferative effect has been demonstrated in human Tenon's capsule fibroblasts. Prevention of posterior capsule opacification is another area for future studies. It is evident that when correct and safe modulation is the objective, alpha-tocopherol merits a concern beyond its mere antioxidant properties.

## Introduction

**Figure 1:**
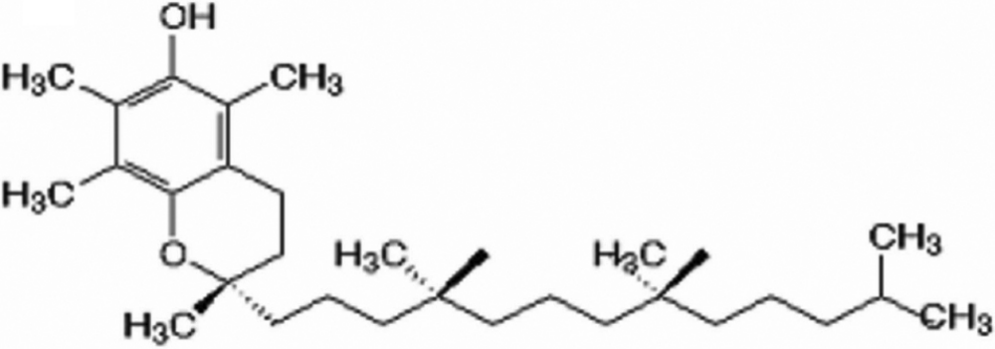
Molecular structure of α-tocopherol. Shown is the molecular structure of α-tocopherol, consisting of two benzene rings with a hydrocarbon chain

Vitamin E is a natural, highly tolerable and cost effective molecule. This generic term is used for tocopherol and tocotrienols consisting of two rings with a hydrocarbon chain. Both structures are similar, although the tocotrienol structure has double bonds on the isoprenoid units. Natural vitamin Es are known as α, β, γ, and δ according to the methyl or proton groups that are bound to their Benzene rings, and the most common and biologically active form is alpha-tocopherol (Figure 1) [1]. When produced synthetically, it is composed of eight stereoisomers in which RRR-α-tocopherol is the most biologically active form [2].

**Figure 2:**
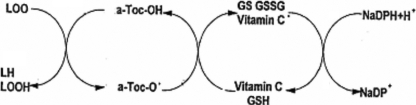
Antioxidant mechanism of tocopherols. LH: Lipid molecule, LOOH: Lipid peroxide, LOO^+^: Lipid Peroxide radical, a-Toc-OH: α-Tocopherol, a-Toc-O^+^: α-Tocopherol radical, GSH: Glutathione, Vitamin C^+^: Vitamin C radical, GS^+^: Glutathione radical, GSSG: Oxidized glutathione, NADPH: Reduced nicotinamide adenine dinucleotide phosphate, NADP+: Oxidized nicotinamide adenine dinucleotide phosphate.

While the recommended daily allowance (RDA) for vitamin E is 8 mg (12 IU) for females and 10 mg (15 IU) for males, Packer [3] recommends up to 1,000–1,200 IU intake of vitamin E in some pathologies including cataract. The principal reserve of natural vitamin E is vegetable oil where its function is to protect tissue from oxidative demage. It is a liposoluble molecule, and, therefore, after dietary intake, vitamin E is not only absorbed easily from the intestinal lumen but is also dispersed between lipids and proteins in cell membranes. Vitamin E molecules can interrupt free radical chain reactions by capturing the free radical. This imparts to them their antioxidant properties. The free hydroxyl group on the aromatic ring is responsible for the antioxidant properties. The hydrogen from this group is donated to the free radical, resulting in a relatively stable free radical form of vitamin E (Figure 2) [2].

Regarding the pharmacodynamics of tocopherols, it has been reported in a study conducted in human eyes that the retinal levels of vitamin E are higher than those of the choroid or vitreous and is correlated with serum levels of vitamin E [4]. It is known that vitamin E can only reach its theraupetic levels in aqueous humor and lens via topical application and is accumulated within the retina when applied via the oral or parenteral route [5]. Moreover, it is reported in animal studies that when 100 mg/kg α-tocopherol is applied via oral or parenteral route, it causes a similar threefold to sixfold increase to its serum levels, though the retinal and vitreal increases are somewhat slower via the oral route [6].

Based on the common knowledge summarized above, vitamin E is occasionally prescribed in ophthalmology clinics. This article provides an overview of the existing literature regarding other effects of this molecule that illuminates the potential usage of this drug as an effective, specific therapeutic tool in several ocular pathologies.

## Discussion

### Beyond the nonspecific antioxidant effect

Specific effects of Vitamin E, which includes gene regulation, have been revealing, and non-antioxidant properties of tocopherols are current topics of interest [7]. In many in vivo and in vitro studies, the antiproliferative effect vitamin E has been shown [8-10].

Protein kinase C (PKC) is one of the pathways used by α-tocopherol [11]. Sharma et al. [12] reported that tocopherol inhibits not only free radical formation but also tyrosine kinase activity in Tissue Plasminogen Activator (TPA)-induced primary human fibroblasts or HL-60 cells. Results of many published in vivo and in vitro collaborative studies illuminate the antiproliferative effect of α-tocopherol via the PKC pathway in the vascular smooth muscle cell model, and they have been summarized by Ozer et al. [13]. In smooth muscle cell cultures, neither the antiproliferative nor the PKC-supressing effects of α-tocopherol have been shown by its isomer, β-tocopherol, or another antioxidant, probucol. It is also shown that, ^3^H Timidin incorporations and PKC activities gradually decline as the α-tocopherol level in the medium rises. On the other hand, linkage of Activator Protein (AP-1) to DNA has been supressed by the application of α-tocopherol in para-methoxyamphetamine (PMA)-stimulated cells, but this effect has not seen in phase G_0_ cells. These findings are also succesfully supported by rabbit studies in the atherosclerosis model.

The existence of sensitive mechanisms to regulate tissue levels is an important and distinctive feature of vitamin E [10,14]. The tocopherol transfer protein, responsible for the intracellular transportation of vitamin E, has been shown and described in cell cultures [15], animals [16-19], and various human tissues [20-22]. Furthermore, discovery of α-tocopherol specific membrane receptors [23] and cytosolic transfer proteins strengthen the thesis that vitamin E possesses properties beyond a mere antioxidant function [10]. Specifically for the eye, scavenger receptor class B type I at the inner blood-retinal barrier has been described in vitro, which is responsible for α-tocopherol uptake from the circulating blood and plays a key role in maintaining α-tocopherol in the neural retina [24].

### Clinical importance

Protective effects of vitamin E have been shown in almost all eye tissues with in clinical, in vitro, and in vivo studies. For instance, vitamin E is known to double the rabbit corneal endothelial cell survival time [25] and enhances retinal cell survival via its effect on mitochondrial activity [26]. Also, α-tocopherol can protect the retina from light injury for up to 24 h of exposure [27]. Vitamin E plays an important prophylactic role against several serious light-induced diseases and conditions of the eye (cataractogenesis and retinal photodeterioration) and skin (erythrocyte photohemolysis, photoerythema, photoaging, and photocarcinogenesis) that are mediated by photooxidative damage to cell membranes [28]. These findings do not have to be explained with antioxidant mechanisms, especially for α-tocopherol. As Azzi [20] stated: “A number of lines of evidence, evolutionary, genetic, biochemical, and functional, have indicated that the natural function of α-tocopherol is that of cell signaling. Such a property is not shared by any other antioxidant molecule.”.

An association between α-tocopherol and some ocular pathologies has also been demonstrated previously. For example, retinitis pigmentosa is shown to be related to an H101Q mutation in the α-tocopherol transfer protein gene [29]. The combination of cryotherapy with vitamin E prophylaxis appeared to decrease the severity and sequelae of threshold retinopathy of prematurity [30]. Average levels of α-tocopherol were shown to be lower in people with exudative macular degeneration [31].

Developments in the understanding of these molecules have increased the attention paid to properties beyond their antioxidant function, including antiproliferative effects. Retinal pigment epithelium cells migrating through the damaged retina play an important role in the pathogenesis of proliferative vitreoretinopathy. Majon et al. [32] found that α-tocopherol inhibits proliferation of human retina pigment epithelium (RPE) cells in culture without exerting cytotoxic effects. Maximal inhibition was achieved with 100 μM α-tocopherol. It has been found that α-tocopherol succinate inhibits proliferation and migration of retinal pigment epithelial cells in vitro [33]. α-Tocopherol and α-tocopheryl acid succinate in saline solution presented a retardation of proliferative vitreoretinopathy in retinal detachments [34].

The protective function of α-tocopherol against the process of cataractogenesis in humans is reported in epidemiologic studies [35]. In the Beaver Dam Eye Study, it is shown that age-related lens opacities in humans are linked inversely to vitamin E status [36].

Glaucoma is another possible area of usage for vitamin E. Failure in glaucoma surgery is primarily due to fibrocellular scar formation, derived from Tenon's capsule fibroblasts. It has been found that d-α-tocopherol (vitamin E) was able to inhibit proliferation of in vitro human Tenon's capsule fibroblasts [37]. Following this, filtran surgery became another model in which an antiproliferative effect has been shown in vivo. α-Tocopherol derivatives showed antiproliferative properties in the experimental models of filtering surgery and showed better intraocular pressure (IOP) control and bleb survival [38,39]. Cell culture studies further illuminated this effect, and comparative studies with other antimetabolites have been performed [40].

On the other hand, dual effects of α-tocopherol and PKC on the eye are of interest in the means of glaucoma therapy. Kunisaki et al. in 1995 [41] and Lee at al. in 1999 [42] showed in different models that retinal vasculer dysfunction due to hyperglycemia could be prevented by α-tocopherol via a diachylglycerol-PKC pathway. In a study performed by Engin et al. [43], 60 glaucomatous eyes from 30 patients were divided into three groups. While group A patients recieved no tocopherol, group B and group C patients were given 300 and 600 mg/day of oral α-tocopheril acetate, respectively. Visual fields and retinal blood flows of ophthalmic and posterior ciliary arteries with Doppler ultrasonography were evaluated in the beginning of the study, as well 6 and 12 months after treatment. Compared with group A, differences of pulsatilty and resistivity indexes of ophthalmic and posterior ciliary arteries were lower in groups B and C 6 and 12 months after treatment. Posterior ciliary artery differences of resistivity indexes in the 6th and 12th months and ophthalmic artery differences of pulsatilty indexes reductions in the sixth month were statistically significant. Differences of mean deviations with visual fields in groups B and C were significantly lower than that of group A.

### Focus on protein kinase C

Among other signaling pathways that have been shown to be affected by α-tocopherol, PKC promises to be an important area of interest. It is a widespread serin/threonine kinase responsible for transduction of the signals taken from the G protein coupled, tyrosine kinase receptors, and nonreceptor tyrosine kinases to the nucleus via phospholipid hydrolysis [44]. c-Jun, c-fos, and c-myc are reported to be the transcription factors that have been activated [45]. Although AP-1 is the mostly considered family [46-48], nuclear factor kappa-light-chain-enhancer of activated B cells (NF-κB) [49] and Transcriptional enhance factor-1 (TEF-1) [50] are among other transcription factors known to be affected by PKC.

Beside its role in retinal vasoregulation mentioned above, the PKC pathway boasts a decisive factor in the pathogenesis for and on the clinical course of glaucoma. Glaucoma is one of the neurodegenerative conditions arising from a compromised glutamate homeostasis. Glutamate transporter activity has been shown to be modulated by PKC [51]. PKC have been shown to affect nonvascular smooth muscle cells such as the iris sphincter [52]. Wiederholt et al. [53] have reported that various pathways and ion channels affect PKC isomers producing different responses in eye nonvascular smooth muscle cells, but in general, PKC inhibitors relax trabecular meshwork while leaving the ciliary muscle comparatively unaffected.

Alexander and Acott [54] have reported that the PKC pathway is crucial in glaucoma therapy for the intraocular pressure-lowering effects of Prostaglandin F 2α (PGF2α) and its analog, latanoprost. The cytokine, Tumor Necrosis Factor α (TNFα), is a strong modulator of trabecular meshwork matrix metalloproteinase (MMP) and tissue inhibitor (TIMP) expressions. TNFα treatment triggered some PKC isoform translocations. Exposure of trabecular cells to TNFα for 72 h differentially downregulated several PKC isoforms. Treatment with a phorbol mitogen that stimulates most PKC isoforms produced strong increases in these MMPs. Effects of TNFα on MMP and TIMP expressions were completely blocked by only one PKC inhibitor.

Further studies were conducted to identify signal-transduction pathways involved. In a study performed concerning cat iris sphincter smooth muscle cells, the relaxing effects of PGF2α and carbachol have been shown to be produced by mitogen activated protein (MAP) kinases in a PKC-dependent manner [55]. On the other hand, PKC activators strongly stimulate the phosphorylation of AQP4 (aquaporin in the ciliary body) and inhibit AQP4 activity in a dose-dependent manner [56].

The prevention of posterior capsule opacification (PCO) is another area for future studies. Despite recent advances in cataract extraction, lens epithelial cells remaining in the capsule proliferate and eventually cause opacification within days of surgery [57]. Although inhibition of lens epithelial cells can be observed with various agents, toxic side effects to the ciliary body, cornea epithelium, and iris limit their use in human subjects [58-60]. PCO is a process mainly involving proliferation and migration of the lens epithelium [61], and PKC is a signaling pathway that is known to result in major effects on this process.

PKC activity exists in the cytosol and particulate fractions of bovine lens epithelial cells [62], and its role in both cell differentiation [63] and proliferation [64] have been shown in rabbit lens epithelial cells. Furthermore, it is shown in lens epithelial cells that, 12(S)HETE-dependent activation of PKCα and β_II_ acts in concert with other epidermal growth factor (EGF)-dependent signals to induce *c-fos* mRNA and that this is independent of the extracellular signal-regulated kinases 1/2 (Erk1/2) pathway [65]. The PKC dependent inositol signaling system also regulates K^+^ fluxes in these highly proliferative lens epithelial cells primarily by affecting the rate via a Na^+^-Cl^-^-K^+^ cotransport mechanism [66]. In addition, PKCγ has a direct or indirect inhibitory effect on gap junction communication in lens epithelial cells [67,68] via phosphorylation of Connexin43 on serine and this causes disassembly and loss of gap junction from the cell surface [69]. PKC also plays a role in cataractogenesis by phosphorylating proteins from calf lens fiber membranes [70] and activating neutral proteases [71]. The PKC-inhibiting effect of vitamin E is known to exist in epithelial cells [72]. Intramuscular vitamin E supplementation is sufficient in protecting histopathologic changes in the lens epithelium [73].

Intracellular functions of vitamin E -beyond its general antioxidant role was an interesting issue even in 1946 [20]. Today, molecular biology is an important discipline to solve curent challenges in ophthalmology. It is already known that certain isomers of vitamin E exert specific effects, and this suggests that proper use of a correctly selected type of vitamin E is likely to provide a significant improvement in the prevention and treatment of many ocular pathologies. Numerous drugs with the potential to manipulate intracellular signal transmission pathways are still being tried. However, both the variations between the receptor and ion-chanel subtypes and the fact that a certain blocker binds to various regions in a condition-dependent manner preclude the production of therapeutics that will bind correctly to the appropriate location. Also, side effects of those substances gravely limit their clinical use. Currently, it is evident that regarding correct and safe intracellular signal modulation, α-tocopherol, a natural, safe, and cost effective drug, merits a careful look beyond its mere antioxidant properties.
